# Gut microbiome influences efficacy of Endostatin combined with PD-1 blockade against colorectal cancer

**DOI:** 10.1186/s43556-024-00200-3

**Published:** 2024-09-10

**Authors:** Jie Xu, Yaomei Tian, Binyan Zhao, Die Hu, Siwen Wu, Jing ma, Li Yang

**Affiliations:** 1 State Key Laboratory of Biotherapy and Cancer Center/Collaborative Innovation Center for Biotherapy, No. 17, West China Hospital, Sichuan University, Section 3, South Renmin Road, Chengdu, 610041 Sichuan The People’s Republic of China; 2 College of Bioengineering, Sichuan University of Science & Engineering, No. 519, Huixing Road, Zigong, Sichuan 643000 The People’s Republic of China; 3 Biological Products Inspection Institute of Sichuan Institute of Drug Inspection, Sichuan, The People’s Republic of China; 4 Frontiers Medical Center, Tianfu Jincheng Laboratory, Chengdu, 610212 China

**Keywords:** Gut microbes, Anti-angiogenic therapy, Adenovirus encoding human endostatin, PD-1 blockade, Cancer therapy

## Abstract

The combination of anti-angiogenic drugs and immune checkpoint inhibitors (ICIs) in the treatment of tumors is emerging as a way to improve ICIs-resistant tumor therapy. In addition, gut microbes (GMs) are involved in angiogenesis in the tumor microenvironment and are also associated with the antitumor function of immune checkpoint inhibitors. However, it is unclear whether gut microbes have a role in anti-tumor function in the combination of anti-angiogenic drugs and immune checkpoint inhibitors for cancer treatment. Endostatin, an angiogenesis inhibitor, has been widely used as an antiangiogenic therapy for cancer. We showed that combined therapy with an adenovirus encoding human endostatin, named Ad-E, and PD-1 blockade dramatically abrogated MC38 tumor growth. The structure of intestinal microbes in mice was changed after combination treatment. We found that the antitumor function of combination therapy was inhibited after the elimination of intestinal microbes. In mice with depleted microbiota, oral gavage of *Bacteroides fragilis* salvaged the antitumor effects of combination Ad-E and αPD-1 monoclonal antibody (mAb) to a certain extent. Further, *Bacteroides fragilis* could improve CD3^+^T cells, NK cells, and IFNγ^+^CD8^+^ T cells in the tumor microenvironment to inhibit tumor growth. Besides, *Bacteroides fragilis* might restore antitumor function by down-regulating isobutyric acid (IBA). Our results suggested that GMs may be involved in the combination of Ad-E and αPD-1 mAb for cancer treatment, which has oncological implications for tumor growth dynamics and cancer immune surveillance.

**Supplementary Information:**

The online version contains supplementary material available at 10.1186/s43556-024-00200-3.

## Introduction

Cancer is caused by the accumulation and mutation of cancer cells in the body [1]. With the continuous development of scientific research, cancer has been proven to be a dynamic process [2], including the interaction between cancer cells and the tumor microenvironment (TME). At present, immunotherapy is known as a method that disrupts the status quo of cancer treatment. It enables cancer patients who cannot be controlled or cured by conventional drug therapy and chemotherapy to have more chances of survival [3]. However, the response rate needs to be largely improved. Accumulating numbers of clinical trials using combination therapies are currently in progress looking for enhanced sensitive methods.

In clinical trials, the number of patients who receive long-term positive feedback from ICIs for cancer treatment remains small due to intrinsic patient factors and induced resistance mechanisms [4]. Therefore, looking for the combination of immune checkpoint inhibitors to treat tumor has become the focus of immunotherapy research [5]. Based on strong theoretical evidence, antiangiogenic agents are considered capable of being used in combination with ICIs for cancer treatment. The occurrence and development of tumor cannot be separated from the formation of abnormal blood vessels, in which vascular endothelial growth factor (VEGF) plays an important role [6]. VEGF promotes the development of abnormal blood vessels, which not only prevents the immune effector from transporting ICIs to the tumorigenic site, but also directly down-regulates the normal anticancer immune function of immune cells [7, 8]. Therefore, anti-angiogenic drugs can improve the efficacy of ICIs at the cellular and molecular levels.

Our laboratory carried out a study on the combination of antiangiogenic drugs and ICIs in the treatment of cancer. Endostatin, a 20-kDa C-terminal fragment of collagen XVIII, is an endogenous inhibitor of angiogenesis [9]. Our previous findings showed that the combination of a recombinant adenovirus encoding human endostatin (Ad-E) and αPD-1 antibody (mAb) improves the anti-tumor efficacy of mouse cancer models, promoting the activated immune response indicated by features such as enhanced T cell infiltration and inhibiting tumor growth.

In addition, GMs are an essential component of the overall ecosystem of human gut microbes that have a normal function. It has been shown that GMs plays a decisive role in the efficacy of PD-1 blockers or CTLA-4 blockers in cancer treatment [10, 11], because GMs and its metabolites can synergistically participate in the activation of immune cells in TME [12, 13]. GMs play an important role in the development of tumors. GMs are essential for the normal functioning of the overall ecosystem of human gut microbes. They can maintain the normal health of the host by regulating the peristalsis of the intestine and producing necessary microorganisms [14]. High levels of *Helicobacter pylori* can promote the formation of gastric cancer [15], while *Pseudomonas aeruginosa* and *Bacillus anthracis* can down-regulate VEGF secretion and inhibit the abnormal formation of tumor blood vessels ultimately [10, 16, 17]. Therefore, GMs play an important role in anti-angiogenic therapy and ICT.

The aim of this research is to highlight the role of gut microbes in the treatment of cancer with antiangiogenic agents combined with ICIs. In this study, we used an antibiotic-induced microbiome depletion mouse model to explore whether the antitumor effects of combination Ad-E and αPD-1 mAb are mediated by the gut microbiome. We demonstrated that microbiota remodeling has an important role in the anti-tumor effects of combination Ad-E and αPD-1 mAb. More specifically, we showed that *Bacteroides fragilis* was important in Ad-E combined with αPD-1 mAb for cancer treatment. After the antitumor effect of the combination therapy was eliminated by the depletion of gut microbiota, *Bacteroides fragilis* increased tumor-infiltrating CD3^+^ T cell, NK cell, IFNγ^+^ CD8^+^ T cells and elevated the level of isobutyric acid (IBA). The current study may have implications for promoting the use of anti-angiogenic drugs in combination with ICIs in cancer treatment, particularly with regard to manipulating gut microbes to try to improve the efficacy of the combination.

## Results

### Combination Ad-E and αPD-1 mAb modified the composition of gut microbiota

**Fig. 1:**
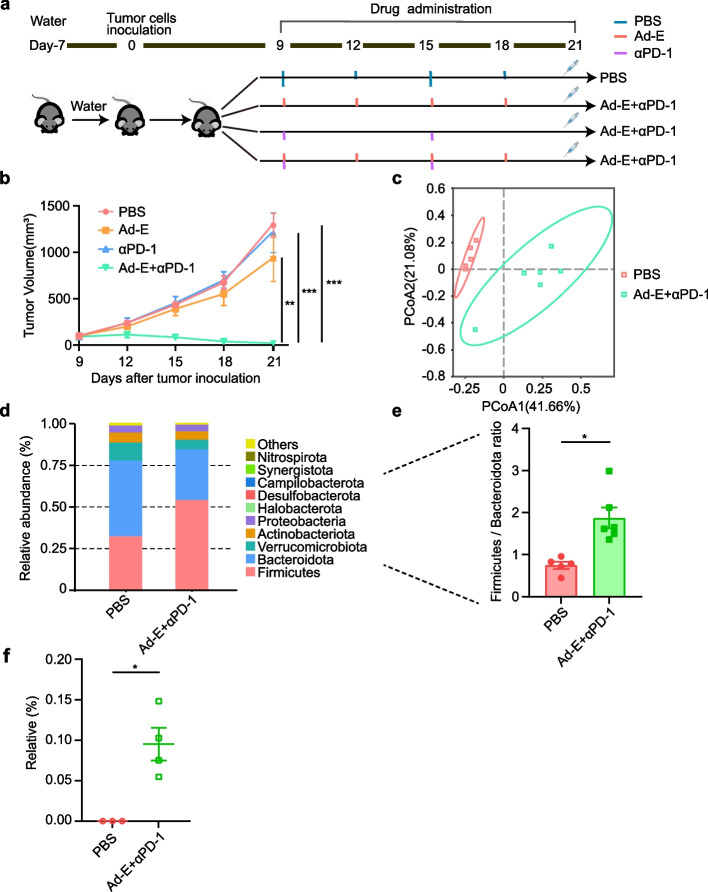
Combination treatment with Ad-E and αPD-1 mAb modulates the composition of gut microbiota. **a** Schematic diagram of the experimental design: mice were inoculated with 1 × 10^6^ MC38 cells, and then treated with PBS, Ad-E, αPD-1 mAb and combination Ad-E and αPD-1 mAb treatment on day 9. **b** Tumor volume of MC38 tumor bearing-mice treated with PBS, Ad-E, αPD-1 mAb and combination Ad-E and αPD-1 mAb treatment, *n* = 5–6 mice. **c** PCoA of fecal samples was performed using Bray–Curtis dissimilarity. Each symbol represents a fecal sample from mice under PBS, Ad-E, αPD-1 mAb and combination Ad-E and αPD-1 mAb treatment, *n* = 5–6 mice. **d** Comparison of phylum-level proportional abundance of feces from mice under PBS, Ad-E, αPD-1 mAb and combination Ad-E and αPD-1 mAb treatment at the end of the experiments. **e** The ratio of *Firmicutes* to *Bacteroidetes*, *n* = 5–6 mice. **f** Relative abundance of *Bacteroides fragilis*, *n* = 5–6 mice. * *p* < 0.05. ** *p* < 0.01. *** *p* < 0.001

Our previous studies have shown that the combination of Ad-E and αPD-1 blockers has a better antitumor effect than the treatment with Ad-E and or αPD-1 blockers alone [18]. To investigate whether the combination of Ad-E and αPD-1 blocker on MC38 tumor-bearing mice has an effect on the intestinal microbiome of mice, we collected mouse feces for 16S rRNA sequencing. Firstly, tumor-bearing mice were divided into four groups as PBS, Ad-E, αPD-1 mAb, combination Ad-E and αPD-1 mAb. Tumor growth was evaluated in untreated-mice and mice under Ad-E, αPD-1 mAb, combination Ad-E and αPD-1 mAb treatment (Fig. 1a). In the group under PBS (control), mice were injected with sterile PBS. The group under Ad-E treatment was injected intratumorally with Ad-E by twice a week. The group under αPD-1 was injected with αPD-1 mAb intraperitoneally once a week. The group under combination Ad-E and αPD-1 mAb treatment was injected intratumorally with Ad-E by twice a week and injected with αPD-1 mAb intraperitoneally once a week. Compared with the PBS group, Ad-E had a weak inhibitory effect on tumor, and PD-1 was almost unable to inhibit tumor growth. Compared with the individual treatment groups, Ad-E combined with PD-1 treatment significantly inhibited tumor growth (Fig. 1b). Since gut microbiota is closely associated with immune checkpoint therapy and tumor angiogenesis, we further examined the effects of combination therapy on gut microbiota.

Next, the fecal matter from untreated and mice under combination Ad-E and αPD-1 mAb treatment were collected and then performed 16S rRNA sequencing on day 21. The principal coordinate analysis (PCoA) revealed significant changes in gut microbiota between mice under PBS and mice under combination Ad-E and αPD-1 mAb treatment (Fig. 1c). At the phylum level, we observed that the relative abundance of *Firmicutes* accounts for the top of the list in PBS group and combination Ad-E and αPD-1 mAb group. Compared with the PBS group, the abundance of *Bacteroidota*, *Verrucomicrobiota* and *Actinobacteriot*a decreased in the combination Ad-E and αPD-1 mAb group (Fig. 1d). Besides, there was little change in the abundance of *Proteobacteria*, *Halobacterota*, *Desulfobacterota*, *Campilobacterota*, *Synergistota*, *Nitrospirota* and other bacteria. In addition, *Firmicutes/Bacteroidetes* (F/B) ratio are emerging as a simple but crude indicator for assessing the gut microbiota. When *Firmicutes* decrease relative to *Bacteroidetes* or *Bacteroidetes* increase relative to *Firmicutes*, it can lead to intestinal flora disorders, chronic inflammation, and digestive system diseases [19, 20]. Our results also showed that compared with the PBS group, the F/B ratio in the Ad-E and αPD-1 mAb combined group was significantly higher (Fig. 1e), indicating that the disorder of intestinal flora had been restored to a certain extent.

To search for biomarkers with statistical difference between the PBS group and the Ad-E and αPD-1 mAb combination groups, we performed LEfSe (LDA Effect Size, the length of the bar chart represents the impact of different species is the LDA score.) statistical tests on the PBS group and combination Ad-E and αPD-1 mAb group at the species level. The results showed that the abundance of *Anaerovorax sp*, *Parabacteroides goldsteinii*, *Lentimicrobium saccharophilum*, *Parabacteroides distasonis*, *Lachnospiracen bacterium* and *bacterium enrichment* were prominent in the PBS group. Besides, Bacteria such as *Prevotella salivae*, *Streptococcus parasanguinis*, *Prevotella melaninogenica*, *Veillonella atypica*, *Actinomyces graevenitzii*, *Sc haalia odontolytica*, *Alloprevotella rava*, *Solobacterium moorei*, *Corynebacterium durum*, *Megasphaera micronuciformis*, *Phocaeico, Bacteroidessus*, *Capnocytophaga gingivalis*, *Bacteroides finegoldii*, *Ruminococcus bicirculans*, *Bacteroides fragilis* were significantly higher in the combination Ad-E and αPD-1 mAb group (Fig. S1). Consistent with this, metaStat analysis showed that the relative abundance of *Bacteroides fragilis* in the Ad-E and αPD-1 mAb combination group was significantly higher than that in the PBS group (Fig. 1f). Overall, we first demonstrated that Ad-E combined with αPD-1 treatment for MC38 mouse tumor model was able to alter the structure and composition of the gut microbiome in mice.

*Bacteroides fragilis*, as one of the least abundant bacteria in human intestinal microbiota, is the most likely to cause intestinal infections [21, 22]. As a pathogen isolated from infected patients and initially named *Bacillus fragilis* [23], *Bacteroides fragilis* is frequently detected in patients with various inflammatory infections, inflammatory bowel disease, endocarditis, bacteremia, and sepsis [24–27]. But recent studies of the relationship between nontoxigenic *Bacteroides fragilis* (B.F.) and the immune system have indicated that several *Bacteroides fragilis* strains may be potential probiotic [28]. However, *Bacteroides fragilis* has been found to potentially have immune-regulating benefits for humans recently. Study has shown that *Bacteroides fragilis* is associated with good anti-tumor immune responses in preclinical tumor models and cancer patients, and can also reduce the incidence of metabolic disorders and various chronic inflammatory pathologies [29].

A recent study has revealed a positive correlation between the proliferation of *Bacteroides fragilis* and the body's anti-cancer immune response. Augmentation with vitamin D leads to an increase in *Bacteroides fragilis*, resulting in the inhibition of tumor growth ultimately [30]. Furthermore, multiple studies have demonstrated that a bacterial polysaccharide (PSA) produced by *Bacteroides fragilis* can modulate the Th1/Th2 balance in mice while augmenting T-cell-dependent immune responses [31, 32]. *Bacteroides fragilis* has been demonstrated to modulate intestinal inflammation by regulating the expression of major histocompatibility complex Class II (MHC-II), toll-like receptor 2 (TLR-2), and tumor necrosis factor-α (TNF-α), as well as the secretion of IL-4, IL-2, and IL-17 in Enteric glial cells [33]. Besides, a previous study in our laboratory found that gavage of mice with *Bacteroides fragilis* ameliorated MTX-induced inflammatory reactions and modulate macrophage polarization [34]. This further indicates that Bacteroides fragilis has a positive immune regulatory function. Therefore, we wanted to explore whether *Bacteroides fragilis* plays an anti-tumor role in the treatment of MC38 mouse tumor model by Ad-E combined with αPD-1 mAb.

### The gut microbiota influences the antitumor effect of combination Ad-E and αPD-1 mAb treatment

To investigate whether the anti-tumor function of the combination of Ad-E and αPD-1 mAb in the treatment of tumors is affected by gut microbes, we conducted the study by using antibiotics to eliminate gut microbes. Firstly, mice were randomly divided into two batches and then pretreated them with PBS or antibiotics (ATB) for a week. Next, mice were injected MC38 cells subcutaneously. When tumor volume reached 50-100mm^3^, mice in each batch were randomly assigned to two groups (PBS and Ad-E and αPD-1 mAb combination treatment). In the PBS group, mice were injected intraperitoneally sterile PBS once a week and injected intratumorally sterile PBS twice a week. In the same way, mice under combination Ad-E and αPD-1 mAb treatment was injected with αPD-1 mAb intraperitoneally once a week and injected intratumorally with Ad-E by twice a week. Thees drugs was administered for two weeks throughout the experiment.

**Fig. 2:**
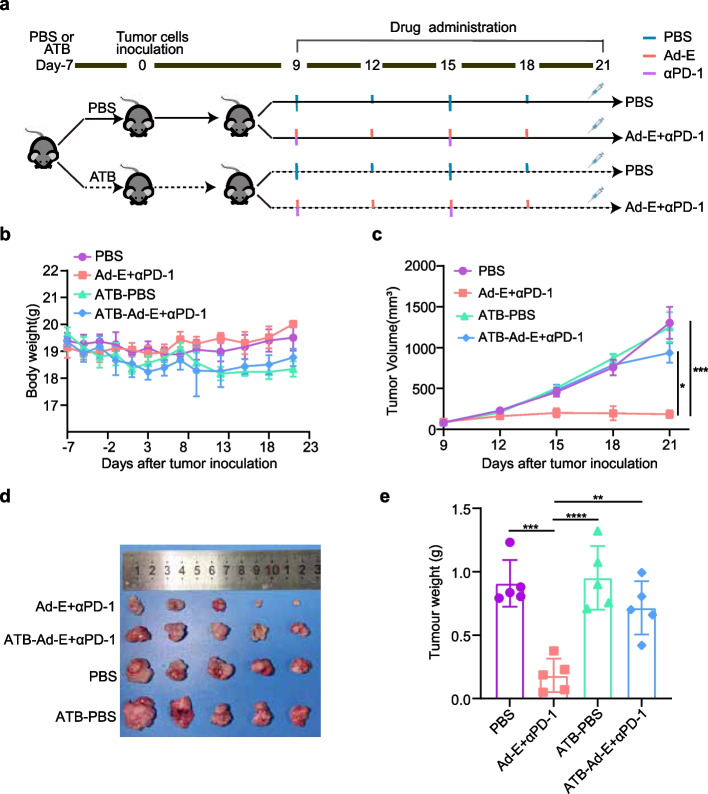
The effect of the combination of Ad-E and αPD-1 mAb on tumor was affected by gut microbiota. **a** Schematic diagram of the experimental design: mice were treated with ampicillin, polymyxin, metronidazole, streptomycin (antibiotics, ATB) or PBS, and they were inoculated with 1 × 10^6^ MC38 cells. Next, mice that treated with antibiotics or PBS were treated by PBS or Ad-E combined αPD-1 mAb on day 9. **b** Body weight changes of mice in the following four groups: PBS (sterile PBS pretreatment, treated with sterile PBS), combination Ad-E and αPD-1 mAb (sterile PBS pretreatment, treated with Ad-E and αPD-1 mAb), antibiotics-PBS (antibiotic pretreatment, treated with sterile PBS), antibiotics-combination Ad-E and αPD-1 mAb (antibiotic pretreatment, treated with Ad-E and αPD-1 mAb), *n* = 5. **c** Tumor growth kinetics of mice were shown in groups, *n* = 5 mice per group. **d** Representative images of dissected tumors on day 21, *n* = 5. **e** Tumor weights of four groups on day 21, *n* = 5. * *p* < 0.05. ** *p* < 0.01. *** *p* < 0.001

Studies have shown that antibiotic-induced microbiome depletion (AIMD) can alter metabolic homeostasis [35]. In our study, both PBS and combination Ad-E and αPD-1 mAb increased the body weight of mice during the 21 days period physiologically (Fig. 2b). Compared to the PBS and Ad-E + αPD-1 groups that mice with gut microbiota, mice without gut microbiota treated with PBS (ATB-PBS) and mice without gut microbiota treated with Ad-E and αPD-1 mAb (ATB-Ad-E + αPD-1) lost body weight on day 9. With subsequent treatment, the weight of mice in the ATB-PBS and ATB-Ad-E + αPD-1 groups increased physiologically before the experimental endpoint. (Fig. 2b).

Consistent with previous studies, combination Ad-E and αPD-1 mAb treatment appreciably suppressed tumor growth, compared with untreated- mice (Fig. 2c). And the tumor volume image of Ad-E combined PD-1 group was smaller than that of PBS group (Fig. 2d). Thus, the final tumor weights of mice in Ad-E combined αPD-1 group were lower under the PBS group (Fig. 2e). In untreated mice, treatment with antibiotics had little effect on tumor growth. Notably, this antitumor effect was significantly diminished in the mice in the Ad-E and αPD-1 mAb treatment group without gut microbiota. Tumor volume was a significant significantly increased in Ad-E combined αPD-1 with antibiotics group compared with Ad-E combined αPD-1 treatment group (Fig. 2c). And the tumor volume image in Ad-E combined αPD-1 with antibiotics group was bigger than that in Ad-E combined PD-1 group (Fig. 2d). Mice in the Ad-E and combined αPD-1 groups without gut microbiota had significantly higher final tumor weights than those in the Ad-E and αPD-1 groups (Fig. 2e), which suggesting that gut microbiota may influence the antitumor effect of combination therapy with Ad-E and αPD-1 monoclonal antibody. This further confirmed that the anti-tumor function of Ad-E combined with αPD-1 in the treatment of MC38 mouse tumor model is related to intestinal microbes.

### *Bacteroidesfragilis* rescues the antitumor effect of combination Ad-E and αPD-1 mAb treatment

**Fig. 3:**
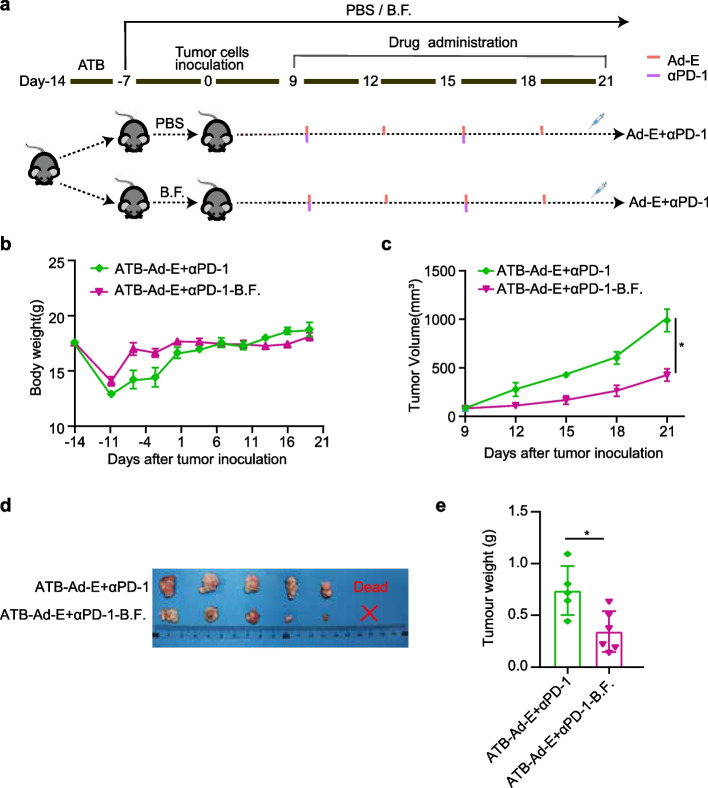
*Bacteroides fragilis* rescues the antitumor effect of combination Ad-E and αPD-1 mAb treatment. **a** Schematic diagram of the experimental design: mice were treated with antibiotics for a week and then evenly divided into two groups. Mice in one group were given PBS, and mice in the other group were given *Bacteroides fragilis*. A week later, both groups of mice were injected with 1 × 10^6^ MC38 cells and given the combination treatment on day 9 after injection. **b** Body weight changes of mice in the following two groups: antibiotic-combination Ad-E and αPD-1 mAb (mice have given antibiotic pretreatment, gavaged by PBS, and treated with Ad-E and αPD-1 mAb), antibiotic-combination Ad-E and αPD-1 mAb-B.F. (mice have given antibiotic pretreatment, gavaged by *Bacteroides fragilis*, and treated with Ad-E and αPD-1 mAb antibiotic), *n* = 6. **c** Tumor growth kinetics of mice were shown in groups, *n* = 6. **d** Representative images of dissected tumors on day 21, *n* = 6. **e** Tumor weights of two groups on day 21, *n* = 6. (Dead: the mice died due to tumor invasion and could not peel off the mouse tumor at the end of treatment. × : the mice tumors disappeared due to effective treatment at the treatment endpoint) * *p* < 0.05. ** *p* < 0.01. *** *p* < 0.001

To verify whether *Bacteroides fragilis* is sufficient to salvage the antitumor effects of combined Ad-E and αPD-1 mAb treatment in the absence of the gut microbiome, we performed a supplemental experiment with *Bacteroides fragilis* in mice with depleted gut microbiota. As shown in Fig. 3a, after the mice were forced oral antibiotics for gut microbiota pre-depletion for 7 days, mice were divided into two groups on average. One group received daily oral gavage of *Bacteroides fragilis*, while the other group received daily oral gavage of sterile PBS. The mice were injected with MC38 cells on the seventh day after the start of gavage. Nine days after the mice were inoculated with MC38 cells, the first administration began when the tumors had grown to an appropriate size. All mice were injected intratumorally with Ad-E by twice a week and injected with αPD-1 mAb intraperitoneally once a week.

At the beginning of the study, pretreatment with antibiotics caused the mice to lose weight. After the pre-depletion of intestinal microorganisms, the weight of mice supplemented with *Bacteroides fragilis* began to recover slowly compared with the control group (Fig. 3b). As the experiment progressed, there was no significant difference in body weight between the two groups of mice and physiologically increased body weight in both groups at the end of the experiment (Fig. 3b). Compared with mice under ATB-Ad-E + αPD-1 group, the tumor growth of mice in ATB-Ad-E + αPD-1-B.F. group progressed slowly. At the treatment endpoint, the size of mice in ATB-Ad-E + αPD-1-B.F. group decreased significantly compared to ATB-Ad-E + αPD-1. (Fig. 3c). And the tumor volume image in ATB-Ad-E + αPD-1-B.F. group was smaller than that in ATB-Ad-E + αPD-1 (Fig. 3d). Moreover, the tumor weight of mice in ATB-Ad-E + αPD-1-B.F. group was significantly smaller than that in ATB-Ad-E + αPD-1, which suggesting *Bacteroides fragilis* was able to reduce tumor weight in tumor-bearing mice at the end of the experiment (Fig. 3e).

In this part of the experiment, we used the control variable method to confirm the important role of *Bacteroides fragilis* in the treatment of MC38 mouse tumor model by Ad-E combined with αPD-1. After the anti-cancer function of Ad-E combined with αPD-1 was inhibited by antibiotic treatment, the supplementation of *Bacteroides fragilis* could restore the anti-tumor effect of the combination to a certain extent.

### *Bacteroidesfragilis* inhibited tumor growth by up-regulating CD3^+^ T cells, NK cells and IFN-γ^+^CD8^+^ T cells.

Recent studies have shown that the gut microbiota can regulate antitumor immunity [36–38]. To further investigate the effect of antibiotic treatment on immune-infiltrating cells in MC38 mouse tumor microenvironment treated by Ad-E combined with αPD-1 monoclonal antibody, we performed flow cytometry to characterize the phenotypes and cytokine profiles of immune cells in the spleen, mesenteric lymph node (MLN) and tumor infiltrating lymphocytes (TILs). Firstly, flow cytometry was performed on mice under PBS (sterile PBS pretreatment, treated with sterile PBS after tumor established), Ad-E + αPD-1 (sterile PBS pretreatment, treated with Ad-E and αPD-1 mAb after tumor established), ATB-PBS (antibiotic pretreatment, treated with sterile PBS after tumor established), ATB-Ad-E + αPD-1 (antibiotic pretreatment, treated with Ad-E and αPD-1 mAb after tumor established). These experimental treatments from mice under different groups are described in detail above.

**Fig. 4:**
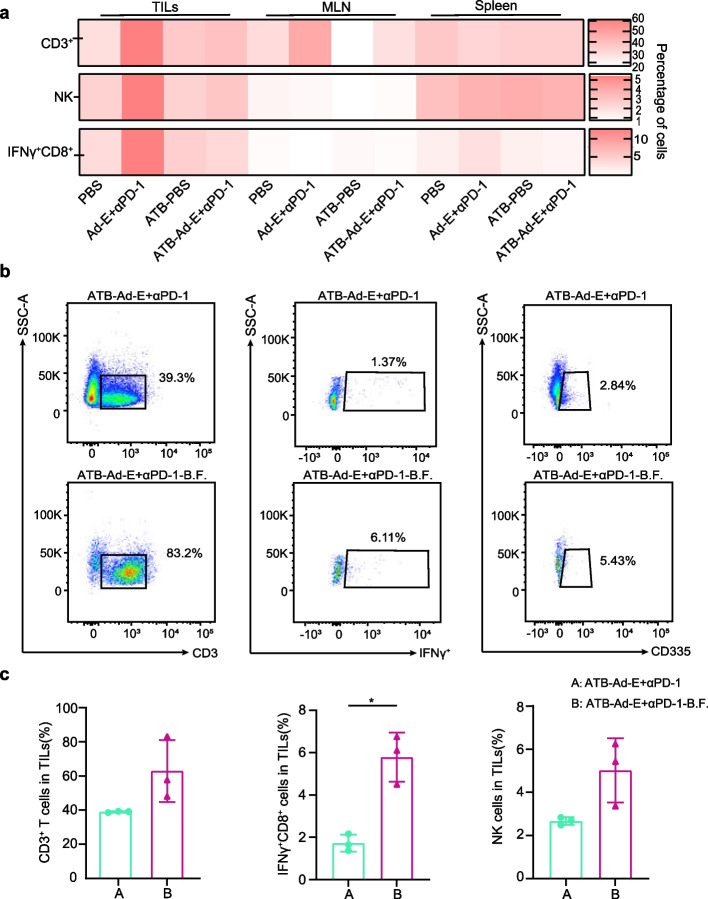
Combination Ad-E and αPD-1 mAb treatment increases CD3^+^ T cells, NK cells and IFN-γ^+^CD8^+^ T cells in a gut microbiota-dependent manner. **a** Flow cytometry showed major changes in immune cells in mouse tumor, MLN, and spleen tissue. The heatmap represents the differences in immune cell and cytokine profiles. Red represents the percentage of immune cells; the deeper the color, the greater the percentage of immune cells. PBS (sterile PBS pretreatment, treated with sterile PBS), combination Ad-E and αPD-1 mAb treatment (sterile PBS pretreatment, treated with Ad-E and αPD-1 mAb), antibiotics-PBS (antibiotic pretreatment, treated with sterile PBS), antibiotics-combination Ad-E and αPD-1 mAb treatment (antibiotic pretreatment, treated with Ad-E and αPD-1 mAb), *n* = 3. **b** Representative flow cytometry plots for one mouse per group in *Bacteroides fragilis* experiments are shown (from left to right: CD3^+^ T cells, IFN-γ^+^CD8^+^ T cells, NK cells). **c** The percentage of tumor-infiltrating immune cells in tumors was analyzed after microbial treatment (from left to right: CD3^+^ T cells, IFN-γ^+^CD8^+^ T cells, NK cells), *n* = 3

Flow cytometry analysis showed the numbers of CD3^+^ T cells, NK cells and IFN-γ^+^CD8^+^ T cells in the spleen were not different among the four groups (Fig. 4a). In MLN, the proportion of CD3^+^ T cells in Ad-E combined αPD-1 mAb group was higher than that in PBS group. And NK cells and IFN-γ^+^CD8^+^ T cells in the MLN were not different among the four groups. However, we found that combination Ad-E and αPD-1 mAb treatment increased the percentage of CD3^+^ T cells, NK cells, and IFNγ^+^CD8^+^ T cells in tumor tissues in comparison with the control group (Fig. 4a). But combination Ad-E and αPD-1 mAb with antibiotics treatment significantly decreased the level of CD3^+^ T cells, NK cells, IFNγ^+^CD8^+^ T cells in tumor tissue (Fig. 4a). This also resulted in the inhibition of the anti-tumor function of Ad-E combined with PD-1 for treatment of MC38 mouse tumors after antibiotic treatment.

Next, in order to further investigate how *Bacteroides fragilis* affects immune-infiltrating cells in tumor microenvironment in the treatment of MC38 mouse tumor model by Ad-E combined with αPD-1 monoclonal antibody. On the basis of the previous flow cytometry, we performed flow cytometry on mice under ATB- Ad-E + αPD-1 group and ATB- Ad-E + αPD-1-B.F. group. spleen, MLNs and TILs of three mice in each group were analyzed for antibody labeling and detection after treatment.

We analyzed and found that CD3^+^ T cells, NK cells, IFNγ^+^CD8^+^ T cells were increased in the tumor tissues of these mice under ATB- Ad-E + αPD-1-B.F. compared to that in ATB- Ad-E + αPD-1 group (Fig. 4b-c). Notably, the proportion of CD8 + T cells was significantly upregulated in ATB- Ad-E + αPD-1-B.F. group compared to ATB- Ad-E + αPD-1 group (Fig. 4b-c). However, antibiotic treatment in ATB-Ad-E + αPD-1 group almost completely inhibited the increase of CD3^+^ T cells, NK cells and IFNγ^+^CD8^+^ T cells compared with PBS group as what mentioned before (Fig. 4a). Therefore, the experimental results of this part further prove that *Bacteroides fragilis* can restore the combined anti-tumor effect of Ad-E and αPD-1 through the accumulation of CD3^+^ T cells, NK cells, IFNγ^+^CD8^+^ T cells to a certain extent.

### *Bacteroidesfragilis* reduce tumor angiogenesis, inhibit tumor cell proliferation and promote tumor cell apoptosis

**Fig. 5:**
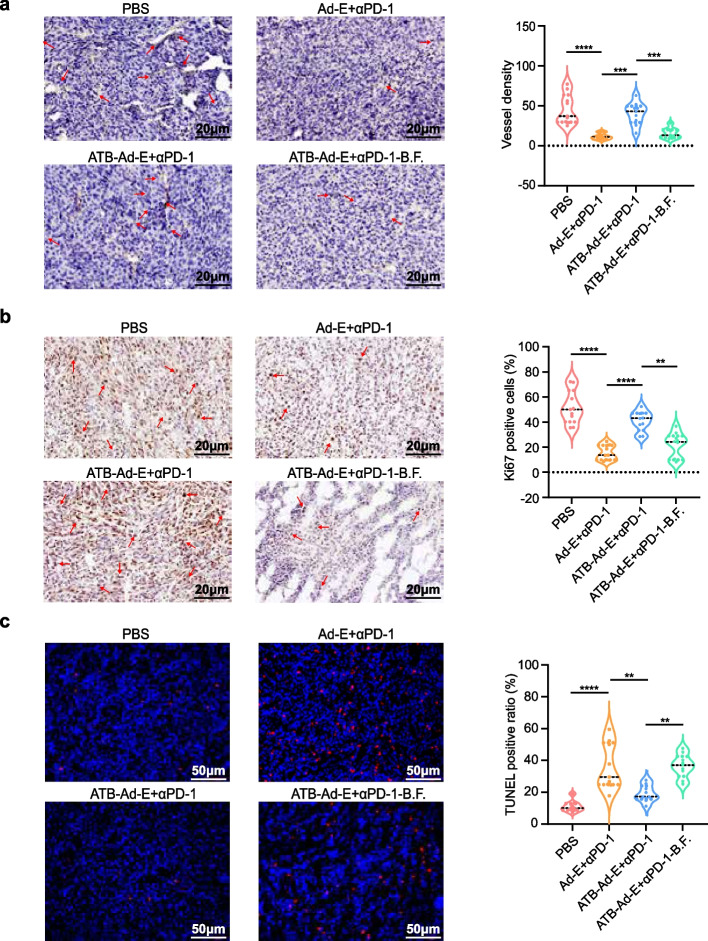
Detection of blood vessels, cell proliferation and apoptosis in MC38 tumor. **a** Vessel density was determined via counting the number of the microvessels per high-power field within hot spot area. 9 high-power fields were counted for each group. Red arrows indicated microvessels. Scale, 20 μm. **b** Representative image of KI67 immunohistochemical staining. The proliferation activity of cells inside the tumor tissue was determined by calculating the number of cells proliferating per high-power field in the hot spot area. 9 high-power fields are calculated for each group. The red arrows represent proliferating cells. Scale, 20 μm. **c** TUNEL staining: Tumor cell apoptosis was detected by in situ TUNEL staining (Red) and DAPI counterstaining (Blue). TUNEL positive ratio was determined by calculating the percentage of apoptotic cells among tumor cells. 9 high-power fields were counted for each group. Scale bars, 50 μm

Tumor tissues of mice in groups PBS, Ad-E + αPD-1, ATB- Ad-E + αPD-1 and ATB- Ad-E + αPD-1-B.F. were used for immunohistochemistry and TUNEL staining. First, in order to observe the distribution of blood vessel density in tumor tissues, CD31 staining was performed on tumor tissues. Consistent with previous studies, the blood vessels in the tumor of mice in the PBS group were more distributed, while the number of blood vessels in the tumor of mice in the combined Ad-E and αPD-1 mAb group was significantly decreased compare with it (Fig. 5a). However, in the combination treatment group with antibiotic intervention, this phenomenon was significantly inhibited, and the blood vessel density in the tumor tissue of mice was significantly increased (Fig. 5a). Finally, the blood vessel density in the tumor tissue of mice was significantly down-regulated in the ATB- Ad-E + αPD-1-B.F. group, which proved that *Bacteroides fragilis* could reduce the formation of new blood vessels in the tumor of mice. Next, to detect the effect of *Bacteroides fragilis* administration on the proliferative activity of tumor cells in mouse tumor tissues, KI67 staining experiment was performed on mouse tumor tissues. The result showed that the tumor cells in the PBS group and the antibiotics-combination Ad-E and αPD-1 mAb treatment group had stronger difference. Compared with PBS group, the proliferation activity of tumor cells in combination Ad-E and αPD-1 mAb group had decreased significantly in the tumor cells (Fig. 5b). Compared with ATB- Ad-E + αPD-1 group, the proliferation activity of tumor cells in ATB- Ad-E + αPD-1-B.F. group was significantly inhibited (Fig. 5b), indicating that *Bacteroides fragilis* inhibited the proliferation of mouse tumor cells.

In addition, in order to analyze the apoptosis of tumor cells, TUNEL experiment was performed on mouse tumor tissues. The results showed that apoptosis significantly increased in the combination Ad-E and αPD-1 mAb treatment group compared with PBS group, which was consistent with previous research results. However, the intervention of antibiotics also importantly reduced the apoptosis of cells in the tumor. Compare with Ad-E combined αPD-1 mAb group, the number of apoptotic tumor cells in ATB-Ad-E + αPD-1 group increased significantly (Fig. 5c). Fortunately, the apoptosis significantly increased again after the supplementation of *Bacteroides fragilis*. The apoptosis significantly decreased in the ATB- Ad-E + αPD-1-B.F. group compared with ATB-Ad-E + αPD-1 group (Fig. 5c). These results indicate that *Bacteroides fragilis* can promote the apoptosis of tumor cells. To sum up, the vascular distribution, cell proliferation and apoptosis of the mouse tumors were consistent with the early tumor volume and tumor weight monitoring data, which all indicated that Bacteroides fragile could restore the immune function of MC38 mice treated with Ad-E and αPD-1 mAb. Therefore, the staining data of mouse tumor section experiments showed that *Bacteroides fragilis* could inhibit the formation of tumor blood vessels, promote tumor cell growth, promote tumor cell apoptosis, and restore the combined anti-tumor function of Ad-E combined with αPD-1 mAb to a certain extent.

### *Bacteroidesfragilis* may manipulate RACK1 protein to restore anticancer function by down-regulating IBA

Short-chain fatty acids (SCFAs) are crucial metabolites that can serve as an energy source and prevent intestinal epithelial cells and lymphocytes from undergoing autophagy due to nutrient starvation [39]. SCFAs in the host are not limited to the gut; they can also disseminate into the blood and thus communicate with multiple cells in target tissues in a G- protein- coupled receptors (GPCR)- dependent manner or by suppressing histone deacetylases (HDAC) epigenetic activity [40]. Thus, SCFAs may mediate the contribution of the microbiota to cancer immunity. In addition, after reviewing the association between SCFAs and disease in the human Metabolome database (HMDB), we found that acetic acid, propionic acid, IBA, butyric acid, isovaleric acid, valeric acid, 4-methylvaleric acid and hexanoic acid are closely related to the progression of colorectal cancer. To investigate whether the administration of *Bacteroides fragilis* in our study had an effect on these SCFAs in mice, we detected their production in the plasma of animals after experimental treatment using ultra-performance liquid chromatography- mass spectrometry (UPLC/MS).

**Fig. 6:**
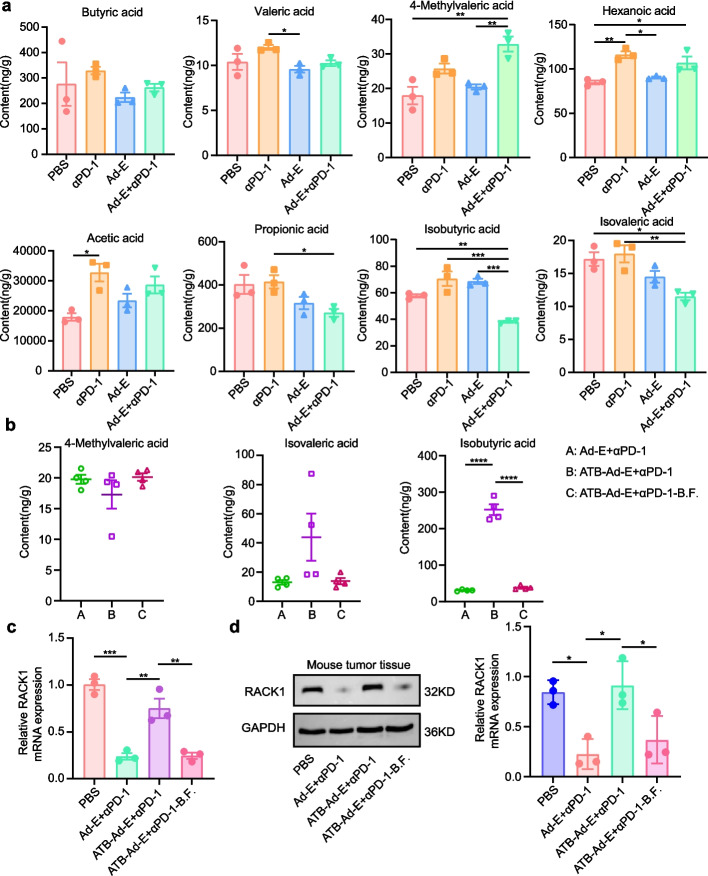
*Bacteroides fragilis* may promote antitumor effects by increasing IBA. **a** Relative abundance of SCFAs in the MC38-bearing model mice treated with PBS, Ad-E, αPD-1 mAb, combination Ad-E and αPD-1 mAb treatment, including butyric acid, valeric acid, 4-methylvaleric acid, hexanoic acid, acetic acid, propionic acid, IBA and isovaleric acid, *n* = 3 **b** Relative abundance of 4-methylvaleric acid, IBA and isovaleric acid, in the MC38-bearing model mice under combination Ad-E and αPD-1 treatment, antibiotic-combination Ad-E and αPD-1 treatment, antibiotic-combination Ad-E and αPD-1 treatment -B.F. groups, *n* = 4. **c-d** RT- qPCR (**c**) and Western blot (**d**) analysis of RACK1 expression in tumor tissue, *n* = 3

First of all, in the previous study, mice were divided into four groups: PBS, Ad-E, αPD-1 mAb, combination Ad-E and αPD-1 mAb. Serum samples were collected at the end of treatment and the levels of these SCFAs were measured. In separate treatments, we found a significant increase of hexanoic acid and acetic acid in αPD-1 group compared to the PBS group (Fig. 6a). Valeric acid and hexanoic acid in Ad-E group was significantly higher than that in αPD-1 group (Fig. 6a). In combination therapy, the results showed that compared to PBS group, the content of 4-Methylvaleric acid increases significantly after combined treatment with Ad-E and αPD-1, while the levels of IBA and isovaleric acid decrease significantly after combination Ad-E and αPD-1 treatment (Fig. 6a). Compared with PBS group, the content of acetic acid increased in Ad-E + αPD-1 group, and the content of propionic acid decreased in Ad-E + αPD-1 group. But there was no significant difference between acetic acid and propionic acid in PBS group and Ad-E + αPD-1 group (Fig. 6a). There was no significant change in other SCFAs between the PBS group and the Ad-E + αPD-1 group (Fig. 6a).

Further, we collected serum from Ad-E + αPD-1 group, ATB-Ad-E + αPD-1 group, ATB-Ad-E + αPD-1-B.F. group. Moreover, content changes of 4-Methylvaleric acid, IBA, isovaleric acid, acetic acid and propionic acid in serum are detected and analyzed. Our data showed the levels of 4-Methylvaleric acid and isovaleric acid did not change significantly in the serum of the three groups of mice. It is worth noting that compared with combination Ad-E and αPD-1 treatment group, IBA content in ATB-Ad-E + αPD-1 group is significantly increased. After the oral gavage of *Bacteroides fragilis*, IBA level is significantly decreased. So IBA in ATB-Ad-E + αPD-1-B.F. group was significantly higher than that in ATB-Ad-E + αPD-1 group (Fig. 6b). In addition, the content of acetic acid did not change significantly after treatment with antibiotics and *Bacteroides fragilis* (Fig. S2). Compared with Ad-E + αPD-1 group, the content of propionic acid increased significantly in ATB-Ad-E + αPD-1 group. It has no significant change in ATB-Ad-E + αPD-1-B.F. group (Fig. S2).

IBA, as one of the end products of intestinal microbiota metabolism, has been reported to significantly increase serum IBA levels in CRC patients compared to healthy individuals and to play a regulatory role in intestinal microbiota CRC proliferation [41, 42]. However, the role and mechanism of IBA in CRC metastasis remains to be explored. Research shows that RACK1 protein is associated with the development of colorectal cancer [43, 44]. Recent studies have shown that IBA promotes colorectal cancer metastasis by activating RACK1, highlighting the key interaction between IBA and RACK1 [45]. Therefore, in order to detect the content of RACK1 in tumor tissues, we conducted RT-qPCR and western blot method. RT-qPCR (Fig. 6c) and western blot method (Fig. 6d) showed that the expression of RACK1 in Ad-E + αPD-1 group significantly decreased because of the antitumor function of drugs compared with the PBS group. In addition, the combined anti-tumor function of Ad-E and αPD-1 was inhibited by the intervention of ATB. Compare with Ad-E combined αPD-1 mAb group, the expression of RACK1 in ATB-Ad-E + αPD-1 group increased significantly (Fig. 5c). However, the supplementation of *Bacteroides fragilis* restored the antitumor function of Ad-E combined with αPD-1 mAb treatment. The content of RACK1 in ATB-Ad-E + αPD-1-B.F. group decreased significantly and recovered to a low level compare with ATB-Ad-E + αPD-1 group (Fig. 6c-d). Therefore, we speculated that *Bacteroides fragilis* might regulate the content of RACK1 protein in tumor tissues by down-regulating the level of IBA in tumor-bearing mice. Finally, the anti-tumor function of Ad-E combined with αPD-1 mAb was restored.

## Conclusion

Intestinal microorganisms and their metabolites constitute the largest microbial ecological environment in the human body. For a long time, the effect of intestinal microbiota on gastrointestinal tumors has been intensely discussed and summarized [46]. In addition, the gut microbiota may participate in the development of gastrointestinal tumors through immune response and secretion of carcinogenic factors [47–51]. As a result, gut microbes have become an emerging target for the treatment of gastrointestinal tumors. There is growing evidence that enhancing the anti-tumor immune response by manipulating gut microbes can promote the development of tumor therapies [11, 38, 52–55]. For example, *Bacteroides fragilis* has been reported that tumors in sterile mice that did not respond to CTLA blocking could be overcome by intragastric administration of *Bifidobacteria fragilis*, polysaccharide immunization with *Bifidobacteria fragilis*, or adoptive transfer of *Bifidobacteria fragilis* specific T cells [54]. In addition, *Bacteroides fragilis NCTC 9343/PSA* can protect against CRC by inhibiting cell proliferation and impairing cell migration and invasion [56]. *Bacteroides fragilis PSA* has a protective effect against lung inflammation [57]. *Bacteroides fragilis ZY—312* improved AAD-associated diarrhea, recovery of intestinal barrier function and intestinal cells regeneration [58].

In this study, we found that *Bacteroides fragilis* was able to promote the tumor suppressive function of combination Ad-E and αPD-1 mAb treatment. Our results demonstrate the role of *Bacteroides fragilis* in vivo anti-tumor activity of Ad-E combined with αPD-1, which could be used for improving therapeutic benefits. In addition, we found changes in the gut microbiome of mice after the combination Ad-E and αPD-1 mAb treatment, but we only studied *Bacteroides fragilis* in depth. Whether other microorganisms play a role in the combination therapy of MC38 mouse tumor models needs further experiments.

Many in vivo studies have shown that the supplementation of intestinal microorganisms is beneficial to the maintenance of immune healthy homeostasis and normal immune function of the host, and also plays an important role in preventing the occurrence and inhibiting the development of cancer [59, 60]. For example, *Lactobacillus rhamnosus* can promote apoptosis by activating Tregs, ultimately playing an anticancer role in breast tumor models [61]. In addition, a mixture of *Lactobacillus paracasei* and *Lactobacillus reuteri* has been reported to reduce the number of mucosal pro-inflammatory cytokines and reduce colitis response in immunodeficient mice [62]. Although studies have also shown that *Bacteroides fragilis* can up-regulate IL-17 secretion by activating Th17 cells, and ultimately promote tumor development [63]. These studies suggest that gut microbes can be involved in tumor development by regulating the function and number of immune cells.

In our experiments involving microbiota, we found that the gut microbes of mice treader by combination Ad-E and αPD-1 mAb were significantly different from those of untreated MC38 tumor-bearing mice. And the abundance of *Bacteroides fragilis* increased significantly in the combined treatment group. Next, antibiotic treatment eliminated the antitumor effects of combination Ad-E and αPD-1 mAb. Interestingly, the antitumor effect of combination Ad-E and αPD-1 mAb treatment in mice was restored to some extent after oral gavage *Bacteroides fragilis* by increasing the accumulation of tumor-infiltrating CD3^+^ T cells, NK cells, IFNγ^+^CD8^+^ T cells. Therefore, our study show that the decrease of intestinal microbiota will weaken the antitumor effect of combination Ad-E and αPD-1 mAb treatment. These data suggest that the antitumor function of combination Ad-E and αPD-1 mAb treatment is related to gut microbes and that *Bacteroides fragilis* enhances the immunomodulatory response to tumors by up-regulating CD3^+^ T cells, NK cells and IFN-γ^+^ CD8^+^ T cells. However, our study focused on the role of GMs in Ad-E combined with αPD-1 therapy, it needs to further investigate whether GMs can influence the therapeutic function of Ad-E or αPD-1, and how the regulation of GMs to Ad-E or αPD-1 up-regulates of combined Ad-E and αPD-1 anticancer function ultimately.

Previous studies have indicated that microbes can produce short-chain fatty acids (SCFAs) to influence the host immune response [64]. In addition, SCFAs derived from the gut microbiota are involved in carbohydrate fermentation (including *Bacteroides fragilis*). And these carbohydrates (such as acetate, propionate and butyrate, etc.) have beneficial functions. They are able to provide energy to the lining of the colon and maintain a stable environment in the colon [65]. Acetate plays a role in maintaining the balance of the mucosal environment by regulating the production of different IgA [66]. Besides, acetate can also promote anti-tumor immunity and contribute to the anti-tumor effect of calorie restriction (CR) [67]. In addition, studies have confirmed that valerate and butyric acid also have important functions in the immunotherapy of cell cancer [68]. To further investigate the antitumor mechanism of *Bacteroides fragilis*, we evaluated the changes in serum SCFAs content of mice after *Bacteroides fragilis* oral gavage supplementation.

Our study showed that *Bacteroides fragilis* was able to up-regulate the level of IBA in serum of mice. However, whether the increase of IBA content is caused by the direct metabolism of *Bacteroides fragilis* needs further study. In addition, a study confirms that the increase of the incidence of colorectal cancer (CRC) and intestinal flora changes associated with a reduced production of SCFAs [69]. Besides, recent studies have also shown that IBA promotes colorectal cancer metastasis by activating RACK1 [45]. Therefore, RACK1 content in mouse tumor tissues was also detected in our experiment. The experimental data showed that the change of RACK1 was also consistent with the detected content of IBA. SCFA is the main microbial fermentation product in the gut [70]. Different SCFAs may play different roles in antitumor immunity. Other SCFAs should be investigated for modulating antitumor immunity under combination Ad-E and αPD-1 mAb therapy.

Taken together, our study shows that the antitumor effect of combination Ad-E and αPD-1 mAb treatment depends on the accumulation of tumor-infiltrating CD3^+^ T cells, NK cells, IFNγ^+^CD8^+^ T cells by gut microbiota. And this effect may be attributed to the production of IBA. Therefore, our results suggest that gut microbiota and SCFAs may promote antitumor immunity and contribute to the combined antitumor effects of anti-angiogenic agents with immune checkpoint inhibitors. Overall, we demonstrated the immunomodulatory potential of gut microbes in anti-angiogenic therapy combined with ICIs in cancer treatment, indicating that the method of manipulating gut microbes to enhance cancer treatment is feasible, which provides ideas for further strengthening anti-tumor therapy.

## Materials and methods

### Animals and cell culture

Female C57BL/6 mice (6 weeks old) were provided by Weitong Lihua Laboratory Animal Limited Company. All mice were fed under specific pathogen-free (SPF) conditions for 7 days before the experiment and treated according to the protocol approved by the Ethics Review Committee for Animal Experiments of Sichuan University.

MC38 colon cancer cells were cultured with Dulbecco's modified Eagle's culture solution (Gibco) containing 10% fetal bovine serum (FBS) (Gibco).

### Recombinant adenovirus encoding human endostatin

As previously mentioned, our laboratory constructed a recombinant adenovirus encoding human endostatin [71]. The recombinant adenovirus was massively amplified in HEK293 cells. The virus was purified by cesium chloride gradient ultrafast centrifugation twice, and the absorbance (A260) was determined by enzyme-labeled apparatus. The titer of the virus was determined by TCID-50 assay for subsequent quantitative experiments.

### Tumor models and treatments

1 × 10^6^ MC38 cells were injected subcutaneously into the right side of C57BL/6 mice to establish a MC38 mouse tumor model. Taking the injection time of tumor cells as the starting point of the experiment, when the tumor volume of mice reached about 50–100 mm3 on the 9th day, the mice were randomly divided into four groups: PBS (treated with sterile phosphate buffer saline, sterile PBS), Ad-E (9 × 10^9^ VP/ mouse), αPD-1 mAb (200 µg/mouse, Bio X Cell) and combination Ad-E and αPD-1 mAb group.

In accordance with the approved treatment regimen, MC38 tumor-bearing mice were injected with recombinant adenovirus expressing endostatin when the tumor grows to the right size once a week for 2 weeks. At the same time, mice were intraperitoneally injected with αPD-1 monoclonal antibody twice a week in the course of a two-week treatment cycle. In addition, the control group will be injected with sterile PBS at the same administration site at the treatment time point. Starting from the successful establishment of the mouse tumor, we measured and recorded the length and width of the tumor every three days, and calculated the tumor volume of the mouse according to the formula:

### Mouse model of depletion of gut microbiota

In order to eliminate the microbiota from the gut of the mice, the mice were fed in SPF conditions for 7 days and then treated with broad-spectrum antibiotics. 6-week-old mice were fed with ATB (non-absorbable antibiotics: ampicillin: 1 g/L; polymyxin: 1 g/L; metronidazole: 1 g/L; streptomycin: 5 g/L) for seven days to deplete the intestinal flora.

### Fecal DNA extraction and 16S rRNA sequencing

We used a Magnetic Soil and Stool DNA Kit (TIANGEN, China) to extract total genomic DNA from collected mouse feces. We treated the DNA and placed it in 1% agarose gel to observe its concentration and purity. The NEB Next® Ultra™ II FS DNA PCR-free Library Prep Kit (NEB) was used to create amplified gene libraries that are quantified by Qubit and Q-PCR. After the library was qualified, NovaSeq6000 was used for PE250 on-machine sequencing. QIIME 2 is used to analyze data in depth.

### Gut colonization with dedicated bacterial species

Mice with or without antibiotic treatment were colonized with 100 µL sterile PBS containing 1 × 10^9^ bacterial cells orally on the day after treatment. *Bacteroides fragilis* was resuscitated and cultured on sheep blood agar plates. After incubation at 37℃ for 48 h, *Bacteroides fragilis* was collected and suspended in sterile PBS. Next, PBS containing *Bacteroides fragilis* was centrifuged and resuspended to approximately 1 × 10^9^ colony-forming units (CFU) per milliliter finally.

### Immunohistochemical staining and apoptosis

At the end of the experiment, the mice were killed by neck removal, and the tumor tissues of the mice were embedded in 4% paraformaldehyde for 48 h.

For immunohistochemical staining, paraffin Sects. (5 μm) of tumor were investigated overnight at 4℃ with either monoclonal rabbit anti-mouse CD31 antibody (1:2000, Abcam) or monoclonal rabbit anti-mouse KI67 antibody (1:2000, Abcam). They were then incubated with HRP-labeled Goat IgG (1:200, Affinity Biosciences). Then, DAB solution (DAB substrate kit, Boster) was incubated to observe the positive reaction.

TUNEL staining was used to evaluate apoptosis in mouse tumor tissues. Following the manufacturer's instructions (TUNEL BrightRed Apoptosis Detection Kit, Vazyme), we stained paraffin Sects. (5 μm) of the tumor using terminal deoxynucleotide transferase (TdT) -mediated tetramethyl rhodamine-deoxyuridine triphosphate (dUTP). Finally, the slices are backstained with DAPI.

### Flow cytometry and antibodies

To get single-cell suspensions, we cut the dissected tumors into small pieces and transferred them into DMEM (Gibco) supplemented with 1.0 mg/mL type I collagenase for 30 min at 37℃. Then, digested tissue suspensions were filtered by 70 µm nylon strainers to remove large fragments of tissue. Mouse spleen and MLNs were isolated and placed in 1640 medium. The spleen cells were incubated in the red blood cell lysis buffer. Then, MLNs and spleen cells were filtered with 100 µm cell filters.

Cell viability and cell surface marker staining was performed at 4 °C away from light at first. Cells were fixed and permeabilized using the Transcription Factor Buffer Set (BD Pharmingen) and then stained with antibodies targeting intracellular markers, according to the manufacturer’s protocol.

For FACS analysis, single- cell suspensions were stained with the following antibodies: Fixable Viability Stain 700, BUV395 anti-mouse CD45, APC-Cy7 anti-mouse CD3e, BV510 anti-mouse CD4, PerCP-Cy5.5 anti-mouse CD8a, BV421 anti-mouse CD335, Alexa 647 anti-mouse Foxp3, FITC anti-mouse CD11b, PE anti-mouse F4/80, BV786 anti-mouse CD86 (all from BD Pharmingen) and BV650 anti-mouse CD206 (Biolegend).

Flow cytometry analysis was analyzed using a FACS Symphony A5 flow cytometer (BD, USA). Data were shown using FlowJo software (V.10.8.1, FlowJo, USA).

### Metabolomic profiling of short-chain fatty acids in the plasma

Blood samples of mice were collected by eyeball blood collection, and serum was obtained from supernatant by centrifugation at 3000 rpm for 20 min. The samples were then derivatized and analyzed using an ultra-high performance liquid chromatography coupled to tandem mass spectrometry (UHPLC-MS/MS) system (Vanquish™ Flex UHPLC-TSQ Altis™, Thermo Scientific Corp., Germany). All of the 11 SCFA standards were obtained from ZZ Standards Co., LTD. (Shanghai, China). Methanol (Optima LC–MS), acetonitrile (Optima LC–MS), ammonium acetate, isopropanol (Optima LC–MS) was purchased from Thermo-Fisher Scientific (FairLawn, NJ, USA). Ultrapure water was purchased from Millipore (MA, USA).

### Quantitative PCR

Total genomic RNA was isolated from mouse tumor tissues using Animal Total RNA Isolation Kit (Foregene) according to its instructions. The proposed RNA is first rigorously tested for purity and integrity testing, then the HiScript III RT SuperMix for qPCR (+ gDNA wiper) (Vazyme) was used to reverse transcription. RT- qPCR was performed using the ChamQ Universal SYBR qPCR Master Mix (Vazyme) to detect RNA expression levels. The relative expression levels were normalized to the expression level of GAPDH and quantified using the 2 − ΔΔCT method. The primers used for RACK1 were F—5′- CGCCACCATGACTGAGCAGAT—3′ and R- 5′- CTAGCGCGTGCCGATGGT- 3′.

### Western blotting

Tumor tissue specimens are ground into a powder in liquid nitrogen at first. Next, the tissue powder is dissolved in a RIPA Lysis Buffer (Beyotime) and protease inhibitor (MCE). The protein concentration was determined using the Enhanced BCA Protein Assay Kit (Beyotime). Proteins were separated by 10% SDS- PAGE gel electrophoresis and transferred onto a PVDF membrane. The membrane was blocked with 5% skimmed milk for 1 h, washed three times with PBST for 5 min each time, and then incubated with primary antibody overnight at 4 °C. The membrane was washed again three times with PBST and then incubated with secondary antibody for 1 h at room temperature. After washing the membrane with PBST three times, the antibody signals were detected using ECL detection reagent (Biosharp).

### Statistical analysis

All data are shown as the mean ± SEM. Differences between groups were analyzed using a two-tailed t test or one-way analysis. Statistical analysis was shown by GraphPad Prism 8.0 (San Diego, California, USA). A P value of less than 0.05 for all data results is considered to be significant.

## Supplementary Information

Supplementary Material 1

Supplementary Material 2

Supplementary Material 3

## Data Availability

Data can be obtained from the authors upon a reasonable request.
