# Supplementary material for: Gut microbiome influences efficacy of Endostatin combined with PD-1 blockade against colorectal cancer
Source: Mol Biomed. 2024 Sep 10;5:37. doi: 10.1186/s43556-024-00200-3 (PMC11383918; doi:10.1186/s43556-024-00200-3)
Supplement: Supplementary file 1 — Supplementary Material 1 [file 43556_2024_200_MOESM1_ESM.docx]

Supplementary Materials for

Gut microbiome influences efficacy of Endostatin combined with PD-1 blockade against colorectal cancer

**Author information**

Jie Xu^1^^#^, Yaomei Tian^1,2#^, Binyan Zhao^1^, Die Hu^1^, Siwen Wu^1^, Jing ma^3^*and Li Yang ^1,4­*^

Author affiliations: 1. State Key Laboratory of Biotherapy and Cancer Center/Collaborative Innovation Center for Biotherapy, West China Hospital, Sichuan University, Chengdu, 610041, China No. 17, Section 3, South Renmin Road, Chengdu, Sichuan 610041, The People’s Republic of China; 2. College of Bioengineering, Sichuan University of Science & Engineering, No. 519, Huixing Road, Zigong, Sichuan 643000, The People’s Republic of China; 3. Biological Products Inspection Institute of Sichuan Institute of Drug Inspection, The People’s Republic of China; 4. Frontiers Medical Center, Tianfu Jincheng Laboratory, Chengdu 610212, China

# These authors contribute equally.

*Correspondence: Jing ma, email: [scidc_bpl@scsyjs.org](mailto:scidc_bpl@scsyjs.org); Li Yang, email: [yl.tracy73@gmail.com](mailto:yl.tracy73@gmail.com)

ORCID

Yaomei Tian: <http://orcid.org/0000-0002-8979-7970>

Li Yang: <http://orcid.org/0000-0002-7880-2113>

**This word file includes:**

Figures. S1, S2 and the full uncropped Blots image(s).

Figure S1


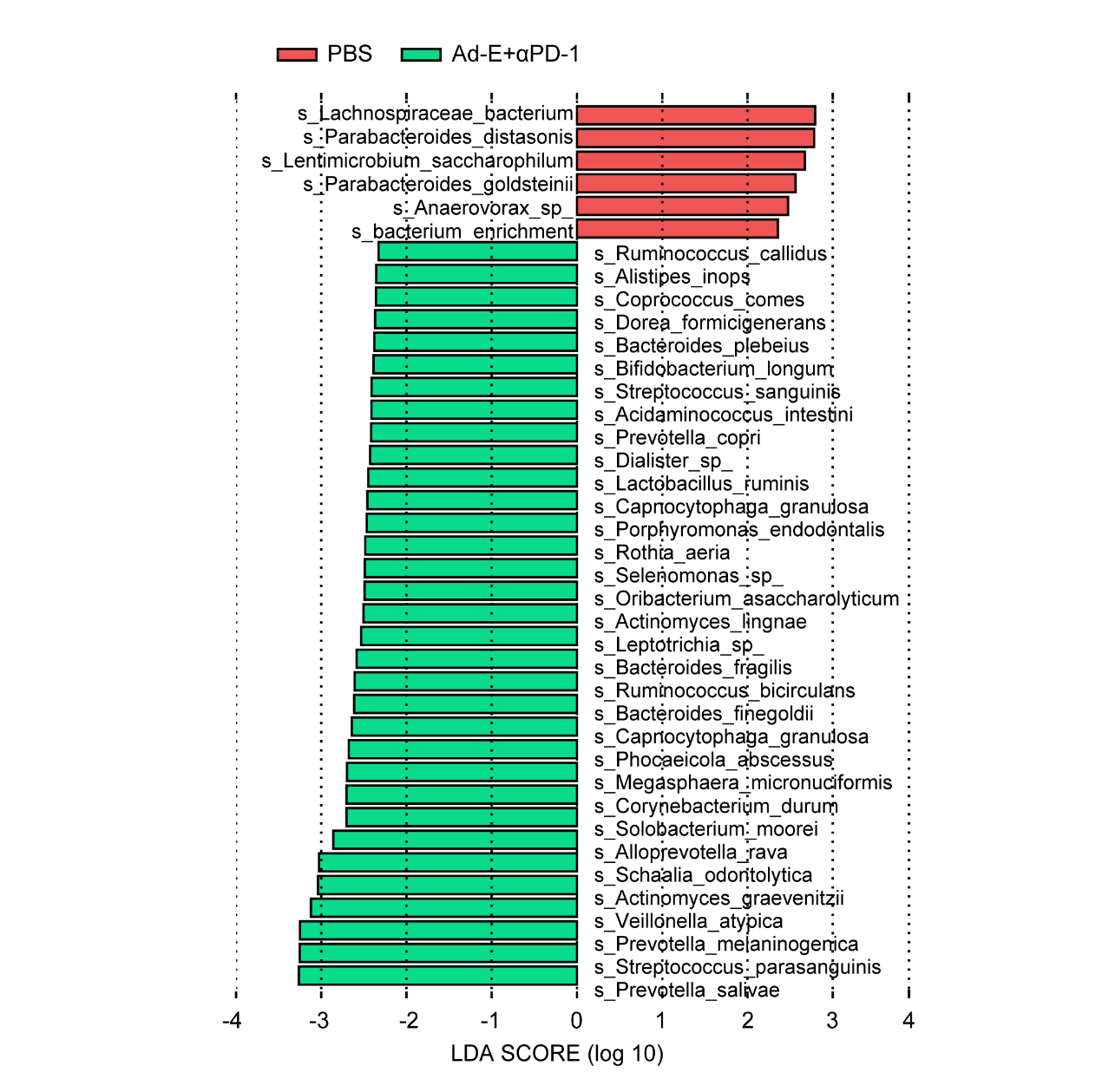


**Fig. S1** LEfSe analysis for differential abundant taxa detected between PBS and combination Ad-E and αPD-1 mAb. LDA score >2.5s, n=5-6.

Figure S2


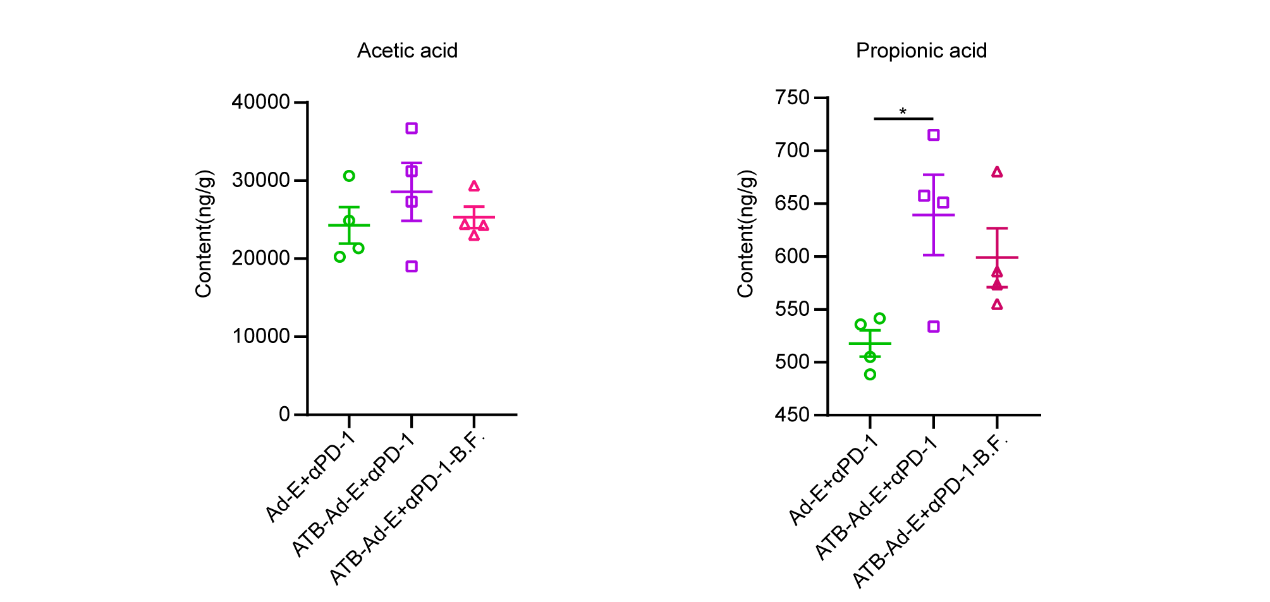


**Fig. S2** Relative abundance of acetic acid and propionic acid, in the MC38-bearing model mice under combination Ad-E and αPD-1 treatment, antibiotic-combination Ad-E and αPD-1 treatment, antibiotic-combination Ad-E and αPD-1 treatment -B.F. groups, n=4.

The full uncropped Blots image(s):

The full uncropped GAPDH images (left) and RACK1 images (left) in Figure 6d (Western blot analysis of RACK1 expression in tumor tissue).
