# Supplementary figures and images for: Gut microbiome influences efficacy of Endostatin combined with PD-1 blockade against colorectal cancer
Source: Mol Biomed. 2024 Sep 10;5:37. doi: 10.1186/s43556-024-00200-3 (PMC11383918; doi:10.1186/s43556-024-00200-3)

PBS Ad-E+αPD-1

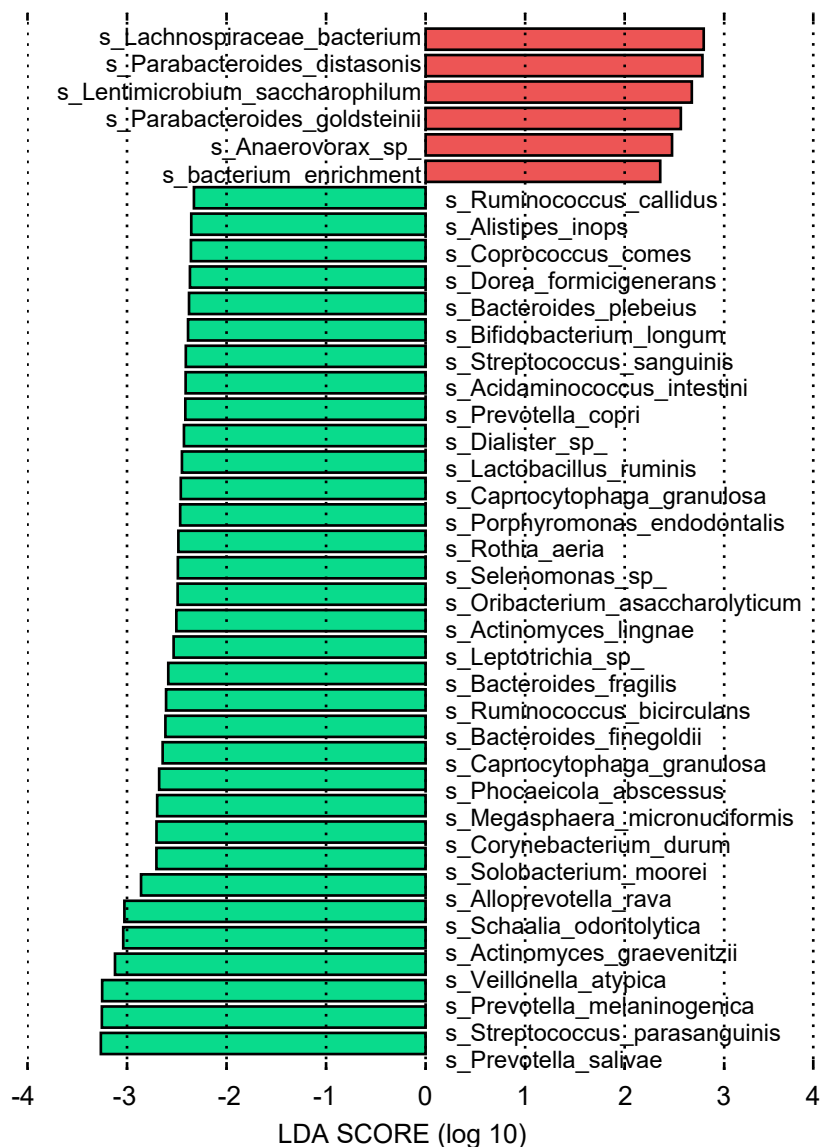

Supplement: Supplementary file 2 — Supplementary Material 2 [file 43556_2024_200_MOESM2_ESM.pdf]

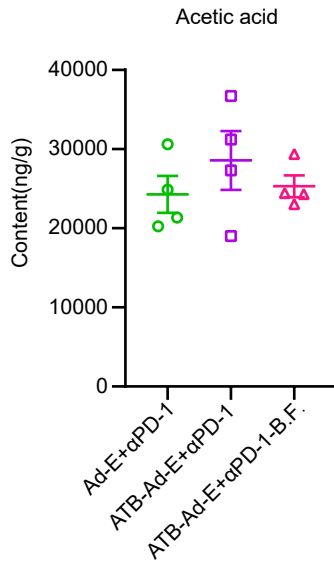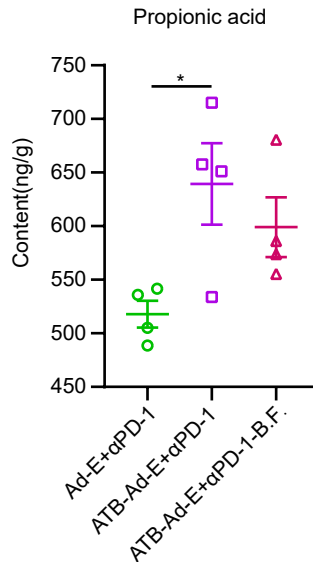

Supplement: Supplementary file 3 — Supplementary Material 3 [file 43556_2024_200_MOESM3_ESM.pdf]
